# Blockchain-enabled wireless communications: a new paradigm towards 6G

**DOI:** 10.1093/nsr/nwab069

**Published:** 2021-04-26

**Authors:** Jiaheng Wang, Xintong Ling, Yuwei Le, Yongming Huang, Xiaohu You

**Affiliations:** National Mobile Communications Research Laboratory, Southeast University, Nanjing 210096, China; Purple Mountain Laboratories, Nanjing 211100, China; National Mobile Communications Research Laboratory, Southeast University, Nanjing 210096, China; Purple Mountain Laboratories, Nanjing 211100, China; National Mobile Communications Research Laboratory, Southeast University, Nanjing 210096, China; Purple Mountain Laboratories, Nanjing 211100, China; National Mobile Communications Research Laboratory, Southeast University, Nanjing 210096, China; Purple Mountain Laboratories, Nanjing 211100, China; National Mobile Communications Research Laboratory, Southeast University, Nanjing 210096, China; Purple Mountain Laboratories, Nanjing 211100, China

**Keywords:** blockchain, wireless communication, 6G

## Abstract

With the deployment of fifth-generation (5G) wireless networks worldwide, research on sixth-generation (6G) wireless communications has commenced. It is expected that 6G networks can accommodate numerous heterogeneous devices and infrastructures with enhanced efficiency and security over diverse, e.g. spectrum, computing and storage, resources. However, this goal is impeded by a number of trust-related issues that are often neglected in network designs. Blockchain, as an innovative and revolutionary technology that has arisen in the recent decade, provides a promising solution. Building on its nature of decentralization, transparency, anonymity, immutability, traceability and resiliency, blockchain can establish cooperative trust among separate network entities and facilitate, e.g. efficient resource sharing, trusted data interaction, secure access control, privacy protection, and tracing, certification and supervision functionalities for wireless networks, thus presenting a new paradigm towards 6G. This paper is dedicated to blockchain-enabled wireless communication technologies. We first provide a brief introduction to the fundamentals of blockchain, and then we conduct a comprehensive investigation of the most recent efforts in incorporating blockchain into wireless communications from several aspects. Importantly, we further propose a unified framework of the blockchain radio access network (B-RAN) as a trustworthy and secure paradigm for 6G networking by utilizing blockchain technologies with enhanced efficiency and security. The critical elements of B-RAN, such as consensus mechanisms, smart contract, trustworthy access, mathematical modeling, cross-network sharing, data tracking and auditing and intelligent networking, are elaborated. We also provide the prototype design of B-RAN along with the latest experimental results.

## Teaser

This work investigates state-of-the-art advances in incorporating blockchain into wireless communications and proposes blockchain radio access network (B-RAN) as a trustworthy and secure paradigm for the future 6G networking.

## INTRODUCTION

The past decades have witnessed an extraordinary upsurge of wireless devices, especially smartphones, along with an exponential growth of diverse wireless traffic [1], which has quickly saturated the capacity of long term evolution (LTE), i.e. the fourth-generation (4G) mobile communication system and accelerated evolution to fifth-generation (5G) wireless networks [2]. To fulfill the enhanced targets, e.g. up to 20 Gbps data rate, 1 million devices/km^2^ connection density and millisecond-level end-to-end latency, 5G adopts a number of key technologies such as an ultra-dense network (UDN) [3,4], massive multiple-input multiple-output (MIMO) [5], millimeter-wave (mmWave) communication [6,7], which have been standardized in the past few years. From 2020, 5G networks are commercially deployed worldwide to provide enhanced mobile broadband (eMBB), massive machine type communications (mMTC) and ultra-reliable and low latency communications (uRLLC) services. At the moment, research of sixth-generation (6G) wireless communications has formally started, aiming to meet requirements of the future in 2030 and beyond. The preliminary studies [8–10] on 6G have reached an agreement that 6G will exceed 5G in its key performance indicators (KPIs) such as spectrum/energy efficiency, connectivity, reliability and latency, and achieve a huge breakthrough compared with the current limited 5G scenarios by providing global coverage via terrestrial, satellite, maritime and unmanned aerial vehicle communications, exploring the full spectrum of sub-6 gigahertz, mmWave, terahertz and optical bands, and supporting a plethora of applications, such as virtual and augmented reality, holographic communications, ultra-high-resolution video streaming, enhanced by big data and artificial intelligence (AI).

The 6G networks will comprise numerous heterogeneous devices and infrastructures to provide ubiquitous wireless connections with ultra-high rates, ultra reliability and extremely low latency and facilitate the Internet of Things (IoT) and the future Internet of Everything (IoE). The increase in scale, density, diversity and complexity in 6G networks brings several significant challenges. One is how to manage enormous resources including spectrum, computation, storage, energy as well as devices and infrastructures and encourage large-scale resource sharing for higher efficiency. Despite the existence of numerous technical solutions for resource management, a major obstacle in reality is the separation between resource hosts or owners, which could be mobile network operators (MNOs), mobile virtual network operators (MVNOs), cloud/edge service providers, resource brokers or even individual users. Usually, they are not willing to make their assets available outside their networks or subnetworks due to the lack of incentives and capital and operating expenditures (CapEx and OpEx). The 6G networking also faces a number of security issues in, e.g. access control, data exchange, privacy protection, certification and so on, which are more demanding and challenging than in 5G given the augmented heterogeneity and density of 6G networks. Such security issues will, in turn, aggravate the separation between networks, subnetworks and their hosts, making efficient resource sharing even harder. Because of these problems, although there have been many efforts made to study highly efficient network resource sharing approaches in history, few of them come into use in practice. One example is cognitive radio (CR) [11,12], which considers letting under-utilized spectra of primary networks be shared by secondary networks. Indeed, lying at the core of the aforementioned issues is *trust* among network entities that is often neglected in network designs. Fortunately and interestingly, the recent blockchain technologies present a promising solution to solve the trust problems in current 5G and future 6G wireless networks.

Blockchain, a generic distributed ledger technology (DLT), has received intensive worldwide public attention as the underlying technical enabler of cryptocurrencies. In 2008, a combination of cryptographic elements was proposed by Satoshi Nakamoto, believed to be a pseudonym, to generate the first successful worldwide payment system known as Bitcoin [13]. In a mere decade, the success of Bitcoin as the first practical cryptocurrency has been remarkable [14,15]. As the backbone of Bitcoin, blockchain was first designed as a growing list of data records that are linked using cryptographic mechanisms. It creates an open and immutable ledger and lends itself to store, secure and manipulate data in a distributed way, while having high credibility in interests protecting and record tracing. In recent years, blockchain has evolved from the original version in Bitcoin to a number of enhanced versions, such as Ethereum [16] and Hyperledger [17], with more functionalities, e.g. being able to support complex program execution via Turing-complete computing. Empowered by its inherent properties such as transparency, anonymity, immutability, traceability and resiliency, blockchain can create trustworthy and secure environments in decentralized manners with low cost and enable a variety of innovative applications and services besides cryptocurrencies. Nowadays, blockchain is widely used in finance and many areas such as logistics, digital voting, tax regulation, copyright protection, health care, to name a few.

Accompanied by the commercial deployment of 5G networks and the study on 6G wireless communications, there has been recent growing interest in incorporating blockchain into wireless communications [8,10]. In particular, at the 2018 Mobile World Congress (MWC), the Federal Communications Commission (FCC) of the United States envisioned the prospect of using blockchain in 6G [18] and emphasized that blockchain will play an important role in wireless networks. Since then, blockchain has been booming in the telecommunication industry. In 2018, AT&T launched edge-to-edge blockchain solutions, aiming to help corporations digitally track business processes throughout the supply chain [19]. Since 2019, T-Mobile has been working with standards bodies and the open source community to develop self-sovereign identity and access management solutions based on blockchain [20]. In China, Alibaba launched the Ant Chain project in 2015 to provide basic blockchain services for communication, data storage and computation [21]. In 2016, Tencent released its first white paper on blockchain that aimed to establish a blockchain-as-a-service platform, namely TBaaS, to provide a one-stop blockchain solution for users [22]. More recently, the China Unicom Research Institute and ZTE corporation proposed several constructive methods of integrating mobile communication systems and blockchain. In 2018, the China Academy of Information and Communication Technology (CAICT) and trusted blockchain initiatives (TBI) proposed the white papers of blockchain technologies and data security for integrating blockchain with the future telecommunication industry.

**Figure 1.**
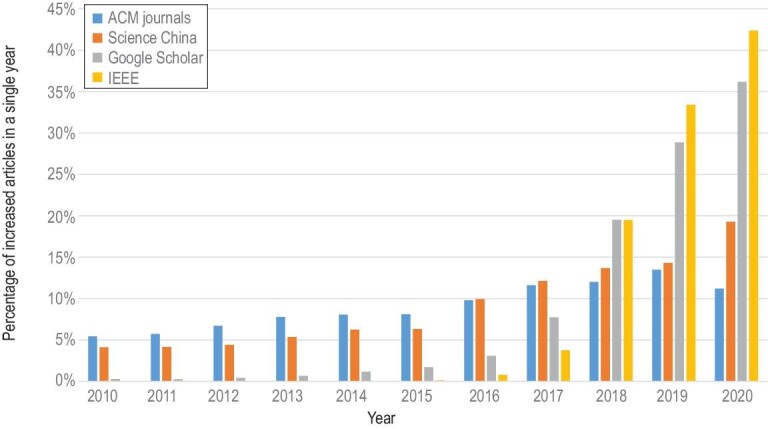
Annual growth rate of academic papers on blockchain in four academic databases (2010–2020).

Blockchain is expected to be the next game changer in the wireless communication area by not only industry but also academia. As shown in Fig. 1, the number of research works related to blockchain in several academic databases has continued to grow in the recent decade. Among them, there are a few pioneering works that attempt to incorporate blockchain into wireless networks. In Ref. [23], an extensive discussion on the opportunities brought by blockchain to empower 5G systems and services was presented. Xie *et al.* [24] provided a survey of blockchain technology applied to smart cities supported by information and communication technology. In Ref. [25], a new concept of the blockchain radio access network (B-RAN) was proposed for future wireless communications and potential functionalities of blockchain applied in resource management and network access were investigated. Liu *et al.* [26] reviewed some efforts on integrating blockchain and machine learning for communications and networking systems and discussed some significant challenges of such integration. Xu *et al.* [27] envisioned the potentials of blockchain for resource sharing in 6G and presented some cases in different application scenarios. So far, most existing research works focus on applying blockchain to one or several specific communication scenarios, for instance spectrum sharing, but there lacks a panorama of comprehensive and deep mergence of blockchain technologies and wireless networks. Meanwhile, critical issues of blockchain, such as security, latency, throughput, scalability, cost and power consumption, have to be investigated for the adaptability and applicability of blockchain in 5G and future 6G wireless networks.

In this paper we aim to present a comprehensive investigation of the most recent advances and challenges in incorporating blockchain into wireless communications, and more importantly establish a unified framework of the blockchain radio access network for upcoming 6G networks as a novel paradigm shift. We first provide a brief introduction to the fundamentals of blockchain, including its concept and architecture, mining and consensus protocols, smart contracts and possible security risks. Then, we provide a comprehensive survey of state-of-the-art works on integrating blockchain into wireless networks from several aspects, including resource sharing, data interaction, access control, privacy protection, tracing, certification and supervision, and highlight the motivations of such integrations and the significant benefits from blockchain.

After summarizing and evaluating the merits and demerits of existing works, we present a unified framework of B-RAN as a trustworthy and secure paradigm for 6G networking by utilizing blockchain technologies. By establishing cooperative trust via blockchain among separated resource hosts as well as heterogeneous network entities, B-RAN facilitates cross-network resource integration and sharing and promises enhanced wireless accessing, roaming, sharing and security across networks or subnetworks. Acting as an open unified framework, B-RAN supports a plethora of services and applications beyond radio access and data transmissions, such as IoT, mobile edge computing (MEC), distributed learning, vehicle networking and energy trading. The underlying blockchain can establish a multifold trust relationship for B-RAN among multilateral groups with any trusted party. In this way, B-RAN pools and shares varied network resources across subnetworks to form a multisided platform (MSP) that leverages the power of positive network effects. In this work, we present B-RAN in a full picture and investigate the application prospects of B-RAN in future 6G networks. We elaborate on the critical elements of B-RAN, including consensus mechanisms, smart contract, trustworthy access, mathematical modeling, cross-network sharing, data tracking and auditing, and intelligent networking. A prototype design of B-RAN and the corresponding experimental results are also provided in this paper.

The paper is organized as follows: In the section entitled ‘Fundamentals of blockchain’ we provide a brief introduction to the fundamentals of blockchain. In the section entitled ‘Blockchain networking’ we provide a comprehensive investigation of state-of-the-art advances in incorporating blockchain into communication networks from several aspects. In the section entitled ‘Blockchain radio access network for 6G’ we present B-RAN as a unified framework of blockchain-enabled wireless communications for 6G networks and elaborate its fundamentals. Finally, we conclude this study in the ‘Conclusion’ section.

## FUNDAMENTALS OF BLOCKCHAIN

In this section, we provide an introduction to the fundamentals of blockchain, including its concept and architecture, mining and consensus protocols and smart contracts, and identify the potential risks of using blockchain in practice.

### Concept and architecture

**Figure 2.**
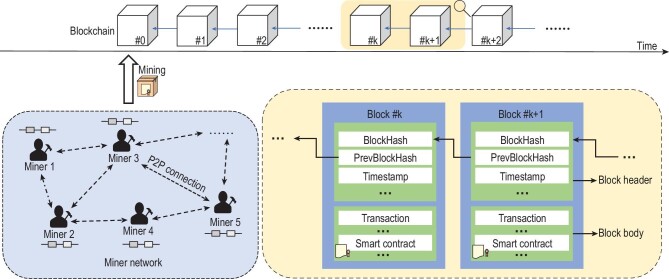
A general architecture of blockchain.

Blockchain is a public database maintained by all active nodes in a peer-to-peer (P2P) network, also known as a distributed ledger [13]. As shown in Fig. 2, it is a chain of linked blocks where a block is an aggregated set of data recording a list of digital actions (e.g. transactions or smart contracts) over time. Each block is identified by a hash value and is interconnected by a hash pointer, i.e. each block contains the hash (hash is a short data digest generated from the data of the block, which varies as the data of the block changes) of the previous block. Because of the mathematical property of hash functions, tampering with any information in a block breaks the links established by the hash pointers. Thus, each newly generated block secures its previous blocks as a confirmation. Generally, it is more difficult to subvert a block that has more confirmations. Blockchain *miners* verify digital actions and group them into blocks on the top of the chain to keep the blockchain consistent and unalterable, often known as mining.

According to data management strategies, blockchains can be classified in two dimensions: public/private and permissionless/permissioned [14]. The main difference between public and private blockchains resides in authentication, i.e. who can access the blockchain. Generally, in public blockchains, anyone can join the blockchain, whereas in private blockchains, the owners control the access to the blockchain. On the other hand, the main difference between permissionless and permissioned blockchains resides in authorization, i.e. who can manipulate the blockchain. Usually, in permissionless blockchains, anyone can update data in the blockchain, whereas only authorized entities are allowed to participate in permissioned blockchains. Notably, consortium blockchains are permissioned and semi-private. Compared with a private blockchain that is commonly controlled by a single owner, a consortium blockchain is jointly maintained by controlled nodes that are from multiple organizations or individuals. It can be regarded as a partially decentralized blockchain that can be autonomous and controllable while assuring the overall security, and it is a suitable option for dealing with multilateral affairs and establishing trust between multiple parties. In practice, an appropriate type of blockchain is carefully selected according to the specific applications.

### Mining and consensus

Consensus mechanisms refer to the protocols used to achieve agreement on the state of blockchain among multiple entities and are vital to blockchain. In this subsection, we survey various consensus mechanisms classified into two categories [28], as shown in Box 1.

Box 1. **Summary of surveyed consensus mechanisms.**

**Table:** 

Proof-based consensus mechanisms	Proof of work [13]	Cuckoo cycle [29] Useful proof of work [30] Proof of learning [31]
	Proof of stake	Peercoin [32] Proof of luck [33] Ouroboros [34]
	Others	Proof of device [25] Proof of human [35] Proof of negotiation [36] Bitcoin-NG [37]
Voting-based consensus mechanisms	Crash fault tolerance	Paxos [38] Raft [39]
	Byzantine fault tolerance	PBFT [40] Proof of authority [41]

#### Proof-based consensus mechanism

Proof-based consensus mechanisms require a miner node to prove that it is more capable than others to append a new block to the blockchain. First invented in Ref. [42], proof of work (PoW) was employed to combat junk e-mails where all participants must finish some work to send an e-mail. The success of Bitcoin [13] made PoW attractive and widespread in academia and the finance industry. PoW-based mining requires miners to solve hash-based puzzles to compete for the right to append a new block. Based on PoW, many studies [29–31] have attempted to utilize the computing capability in PoW for meaningful tasks and avoid wasting power on hash operations. As another line of research, proof of stake (PoS), first proposed in Peercoin [32], measures the ability of miners for block generation based on the miners’ stake [33].

The aforementioned consensus mechanisms can be incorporated into the framework of proof of X (PoX) [14], which requests all nodes within a blockchain network to prove the possession or commitment of certain measurable resources beyond hash operations in a verifiable way. In the framework of PoX, enormous alternative mechanisms have been proposed in terms of cost [25], eco-friendliness [35], fairness [36] and performance [37]. For wireless environments, consensus mechanisms are carefully chosen and designed by considering the resource-costly block distributions and power-limited mining devices. (We refer the reader to the section entitled ‘Consensus mechanisms’ for further discussion.)

#### Voting-based consensus mechanism

Voting-based consensus mechanisms are often adopted in consortium blockchains. Specifically, a block is generated based on the decision of the majority of nodes [28]. In contrast to the PoX relying on weak network connectivity, voting-based mechanisms require fully connected topology among the nodes to validate the blocks and achieve a final agreement.

Among the voting-based mechanisms, crash fault tolerance (CFT) consensus can tolerate the corrupted nodes, which fail to respond to messages due to hardware malfunction, software failure or network disconnection. Paxos [38] and Raft [39] are typical examples of CFT-based consensus. However, in blockchain, there may exist Byzantine nodes that behave unpredictably. Lamport *et al.* [43] pointed out that all nodes could reach a final decision only if the number of nodes is strictly greater than three times the number of Byzantine nodes, which is well known as the Byzantine generals problem. The most famous Byzantine fault tolerance (BFT) mechanism is the practical Byzantine fault tolerance (PBFT) [40]. PBFT relies on a leader-peer hierarchical structure for block proposal and utilizes a three-phase process to reach a final decision for all the nodes. Moreover, it provides the properties of low latency, high throughput and low-energy consumption, but is subject to poor scalability due to the high communication complexity. Besides, as an enhancement to the BFT mechanism, proof of authority (PoA) [41], adopted by Ethereum, is based on the identities of network entities. Compared with PBFT, PoA reduces the communication overhead and improves the overall consensus efficiency [44].

### Smart contract

The digital actions in blockchain are taken by *smart contracts*, the scripts in blockchain allowing for the automation of multistep processes. As introduced in Ethereum [45], the concept of smart contract is presented as a cryptographic box, which can only be unlocked when certain conditions are met. Once a smart contract is activated, i.e. being called after having been recorded in the blockchain, the contract terms are enforced and executed automatically among network participants without relying on a third party or a central node. Unlike the unspent transaction outputs (UTXOs) in Bitcoin, the weak version of smart contracts [13], the flexibility and diversity of smart contracts empower blockchain to form a distributed virtual machine beyond a simple cryptocurrency transaction system. The digital assets (e.g. storage, transmission, calculation) and actions (e.g. transactions, fees, interests) can easily be authorized and authenticated by digital signatures and key pairs in smart contracts. With the popularity of Ethereum, smart contracts have become an indispensable component of various emerging blockchains and the functionalities of smart contracts have also been significantly expanded [46].

Despite the benefits of smart contracts, there are security and implementation issues with smart contracts. Several prominent risks of implementing smart contracts were examined in Harris [47], who put forward three key properties (deterministic, isolated and terminable) of a healthy smart contract. Sayeed *et al.* [48] analysed seven attacks that target smart contracts in blockchains and recommended several methods progressing towards a secure smart contract solution. Currently, matured standards of verifying smart contracts, which are vital for securing the digital assets and actions in smart contracts, are still absent and thus needed [49]. Qian *et al.* [50] proposed several embedded analysers and benchmarks to identify defects (e.g. reentrancy and code clone) in smart contracts. In terms of implementation, Parizi *et al.* [51] conducted a comprehensive evaluation of existing domain-specific programming languages of smart contracts for newcomers and researchers, and Pinna *et al.* [52] surveyed some metrics for measuring the attributes (e.g. size and complexity) of smart contracts.

### Potential risks

Similar to common distributed systems, blockchain inevitably suffers from various security attacks. In this section, we summarize the potential risks in blockchain and briefly discuss their features and countermeasures.

#### Alternative history attack

An adversary can launch an alternative history attack for double spending by privately mining an alternative fork, which actually exposes the vulnerability of distributed systems [13]. The adversary can subvert a confirmed chain accepted by the miner network if it is lucky enough to create a longer fraudulent chain. In PoW, if the adversary holds more than 50% of the computational power of the entire blockchain network then it can always alter a confirmed history successfully, namely a 51% attack [13] or Goldfinger attack [53]. Such a powerful attacker can constantly drive miners off, which in turn consolidates the attacker’s position and increases his share. Similar to 51% attacks, the long-range attacks [54] in PoS can alter the history of blockchain and cause inconsistency in blockchains. By collecting the private keys of older accounts that have accrued a majority stake in history, the attacker can construct a fork chain to overlay the current main blockchain. To resist this attack, the authors of Ref. [32] suggested using checkpoints (blocks until which the blockchain is regarded as ‘finalized’ and immutable) to limit the range of long-range attacks.

#### Selfish mining

The key idea of selfish mining is to increase an attackers’ winning probability by letting the honest miners waste power on the public chain [55]. When an attacker finds a valid block, it continues mining the next block without releasing the newly generated block. Until other miners find a valid block, the attacker will publish all blocks previously mined to the network. (Of course, the attacker also bears a great risk that the public chain may overtake its private chain.) Bahack [56] analysed a series of selfish mining strategies and proposed a solution to mitigate the consequences of selfish mining. Bai *et al.* [57] described the state transitions of public and private chains in selfish mining by using the Markov chain model and analysed the profitability of selfish mining when there were multiple selfish mining pools.

#### Cryptanalytic attack

In principle, cryptanalytic attacks (e.g. key attack and quantum attack) in blockchain aim to break the cryptographic algorithm and expose its keys. The blockchain foundation cannot be separated from cryptographic algorithms. For example, Hyperledger Fabric relies on the elliptic curve digital signature algorithm (ECDSA) to generate private keys, but the ECDSA is vulnerable to key attacks [58]. Generally, a key attack happens imperceptibly because of the private key leakage vulnerability and weak randomness of key generation. It was pointed out in Refs. [59,60] that the private keys should be generated by strong randomness to prevent key attacks. On the other hand, the development of quantum computing has a significant impact on traditional cryptographic algorithms as well as blockchain. In Ref. [61], the potential quantum attack in blockchain was investigated and an anti-quantum transaction authentication scheme was proposed.

#### Nothing at stake

Low-cost alternative consensus protocols, such as PoS, are even more vulnerable. If something is at stake, it is at risk of being lost, whereas if nothing is at stake, the adversary has nothing to lose and attempts to launch nothing-at-stake attacks and work on multiple branches simultaneously. That is how a nothing-at-stake attack arises. Buterin [45] recognized this issue and proposed an algorithm, namely Slasher, to prevent this attack by requiring validators to provide a deposit that will be locked for a period. A similar case occurs in the coin-age accumulation PoS, in which an attacker can accumulate coin age by hoarding his coins to increase his influence in the blockchain. Li *et al.* [62] suggested introducing a cap on the coin age to resist this attack.

#### Traditional cyber attack

Traditional cyber attacks, such as the distributed denial of service (DDoS) attack, replay attack, man-in-the-middle attack, Sybil attack and eclipse attack, still exist in blockchain. The DDoS attack occurs when multiple blockchain nodes are flooded with invalid requests and their normal operations may be abruptly interrupted. The replay attack is to intercept the data packets of communicating parties and relay them to their destinations without modification, while in man-in-the-middle attacks, attackers can intercept those data packages and inject new contents. Ekparinya *et al.* [63] presented an example of applying the man-in-the-middle attack to raise double spending in a private Ethereum blockchain. Besides, a malicious entity could create many fake identities to launch a Sybil attack [64] where a plural of faulty information is injected into the network. Unlike Sybil attacks aiming at the entire blockchain network, the eclipse attack only cheats on one network target and forges a false view of blockchains.

## BLOCKCHAIN NETWORKING

**Figure 3.**
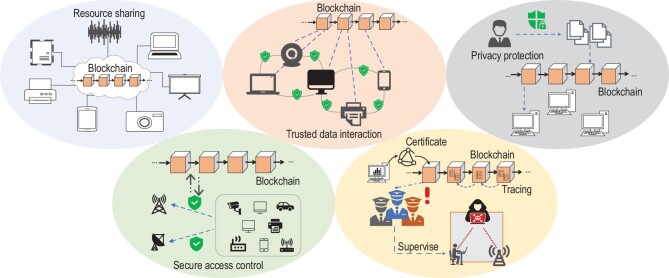
Potential blockchain applications in networking.

As a disruptive technology, blockchain is capable of solving a number of trust and security related problems in communication networks, facilitating more efficient resource sharing, boosting trusted data interaction, secure access control and privacy protection, and providing tracing, certification and supervision functionalities for 5G and future 6G networks as depicted in Fig. 3. In recent literature, there has been increasing efforts to apply blockchain technologies to wireless networks, which will be comprehensively reviewed in this section.

### Resource sharing

The explosive growth of various mobile services demands a large quantity of network resources, e.g. spectra and infrastructures, which are generally limited and have to be shared for better utilization and efficiency [65]. In practice, however, resource sharing is often deterred by the separation between resource hosts, who may lack incentive or have cost and security concerns, making coordination and cooperation between network entities infeasible. On the other hand, with the new functionalities of cloud processing, MEC, software-defined networking (SDN) and network functions virtualization (NFV) in 5G systems, there are increasing types and quantities of network resources, e.g. computing and storage resources as well as network slices, which make resource management and sharing quite challenging. Blockchain and its inherent characteristics can effectively promote collaboration and alleviate the trust and security concerns among separated network entities, thus leading to more efficient resource sharing.

#### Spectrum sharing

There has been intensive research around applying blockchain to spectrum sharing. Weiss *et al.* [66] explored how to implement spectrum management in combination with blockchain and discussed the pros and cons of different spectrum sharing mechanisms. Han and Zhu [67] proposed a spectrum sharing system between operators based on consortium blockchain to provide reliable privacy and security guarantees. In Ref. [68], a blockchain verification protocol was proposed to enable and secure spectrum sharing in moving cognitive radio networks without constant spectrum sensing. Zhou *et al.* [69] proposed a blockchain-empowered spectrum sharing framework that can effectively motivate primary users to share their under-utilized spectrum and realize efficient spectrum allocation with low complexity. Moreover, Fan and Huo [70] used blockchain to construct an unlicensed spectrum management framework for semi-distributed wireless networks and solved the spectrum contention. Maksymyuk *et al.* [71] introduced an intelligent network architecture to deal with the unlicensed spectrum sharing between operators and users via smart contracts. In addition, a new blockchain structure with corresponding consensus algorithms was introduced in Ref. [72] to autonomously manage unlicensed spectrum and decrease the CapEx and OpEx of network deployment.

#### Computing and storage

The wide use of cloud processing and MEC makes computing and storage capacities valuable network resources, which can be efficiently managed by blockchain [73,74]. Chatzopoulos *et al.* [75] proposed a blockchain-based computation offloading framework that enhances the collaboration among entities in sharing computing resources. Liu *et al.* [49] proposed a blockchain-based MEC architecture and used a three-stage Stackelberg game to model service bidding, negotiation and transactions among different entities. In Ref. [76], two double auction mechanisms were utilized to encourage blockchain entities to share their computing power. Furthermore, Wang *et al.* [77] introduced a consortium blockchain for resource transactions in vehicular edge computing and to defend against malicious behaviors. Sun *et al.* [78] utilized blockchain to construct an attribute-based encryption scheme for secure storage and sharing of medical records. In Ref. [79], a blockchain-based arbitrable remote data auditing scheme was proposed to provide reliable network storage services.

#### Infrastructure and device

Blockchain presents a secure and efficient way to manage heterogeneous devices and infrastructures in 5G and IoT networks. Mafakheri *et al.* [80] explored blockchain to fulfill sovereign, autonomous and trusted infrastructure sharing in 5G small cell networks. Dong *et al.* [81] considered using blockchain as a secure, distributed cyberinfrastructure for the future grid and proposed a prototype to optimize energy infrastructure allocation and improve energy efficiency. Huh *et al.* [82] proposed a method to use blockchain to control and configure IoT devices, and attempted the identity management for interconnected devices. Novo *et al.* [83] presented several blockchain-based solutions to mitigate the issues associated with the management of numerous constrained devices. In Ref. [84], a private-blockchain-based architecture for the management and monitoring of IoT devices was introduced. Yu *et al.* [85] constructed a blockchain-based IoT architecture to organize and share IoT data and devices.

#### Network slicing

Enabled by SDN and NFV in 5G systems, network slicing, as logical assembling of diverse physical network resources [86], has an inherent sharing property. Backman *et al.* [87] presented the concept of the blockchain network slice broker to promote slice leasing, and later in Ref. [88] the feasibility of blockchain network slice brokering was analysed in an industrial automation scenario. Zanzi *et al.* [89] proposed a novel network slicing brokering solution named NSBchain, which enables infrastructure providers to allocate network resources to the intermediate brokers through smart contracts. Similarly, Togou *et al.* [90] designed a signaling-based distributed on-demand framework called distributed blockchain-enabled network slicing that promotes dynamic resource leasing between different service providers to support high-performance end-to-end services.

### Trusted data interaction

With the upsurge of diverse wireless traffic and connection density, data from varied sources need to interact and collaborate to provide services together [91]. However, the lack of trusted relationships among data holders participating in the mobile network makes it difficult to secure data interaction processes and verify data authenticity and reliability [92]. Recently, researchers have been using blockchain to establish mutual trust between diverse devices and create a trusted channel for secure data interactions [92,93]. The efforts of using blockchain to support trusted data interactions in wireless networks have mainly been in two directions: to ensure the credibility of each network identity and to improve the authenticity of the transmitted data.

#### Identity credibility

To ascertain identity credibility, each entity can obtain its credibility value before entering the network by letting blockchain participants analyse a number of indicators (e.g. its historical behaviors), and then the permissions will be granted based on the evaluated value to the entity. In the trust management mechanism in Ref. [94], only the nodes with a specific trust degree can interact with other nodes, while malicious nodes will be detected and expelled. Chai *et al.* [95] designed a consensus mechanism for blockchain-based V2X networks, in which the validation of data interaction is conducted based on vehicles’ credit degrees. In addition, by combining distributed identities with the underlying layer of blockchain, Shi *et al.* [92] managed to enhance personal privacy and control of digital identities. Moreover, Javaid *et al.* [96] proposed a trustless system model for intelligent vehicles using blockchain and a certificate authority in vehicular ad-hoc networks.

#### Data authenticity

Group intelligence perception and consensus mechanisms could be utilized together to ensure the accuracy and authenticity of the data. Ma *et al.* [97] took advantage of the mutual authentication protocol and user-defined sensitive data encryption in a blockchain-based trusted data management scheme in edge computing. Yang *et al.* [98] proposed a decentralized trust management system for vehicular networks based on blockchain by using a Bayesian inference model to evaluate the credibility of traffic messages. Also, Yang *et al.* [99] proposed a proof-of-event consensus concept for vehicular networks that uses passing vehicles to verify the authenticity of traffic data collected by roadside units.

### Secure access control

The continuous densification of wireless networks and increasing heterogeneity of massive devices bring many security risks to access control in mobile communication systems. Specifically, there are mainly three categories of security risk: device security risk caused by malicious device intrusion, system security risk due to the single point of failure and data security risk resulting from data leakage. Built on its inherent nature, such as tamper resistance, decentralization and fine-grained auditability, blockchain presents a promising remedy to address these security risks in wireless networks.

#### Device access control

Given the massive number of various devices in the mobile communication network, there are inevitably malicious devices attempting to compromise the security of the system. Several works have considered using blockchain to prevent malicious device intrusion [100–102]. Javaid *et al.* [100] adopted a customized smart contract to defend against DDoS attacks and rogue device attacks. Pinno *et al.* [101] designed an access control architecture called ControlChain, which provides a secure way to create relationships for network entities and assign them attributes. Moreover, Zhang *et al.* [102] proposed an access control framework containing three smart contracts to safely add, update and delete network entity identities.

#### System access control

In addition to malicious device intrusion, the traditional access control mechanisms also face the risk of single points of failure due to the fact that they are based on centralized entities. The characteristics of decentralization and joint maintenance in blockchain can readily prevent the single point of failure. Some researchers have tried to integrate blockchain with access control mechanisms to solve this concern [82,83,103,104]. Ding *et al.* [103] proposed an attribute-based access control scheme for IoT, which utilizes blockchain to record the distribution of attributes to avoid single points of failure and data tampering. Moreover, Xu *et al.* [104] devised an identity-based robust capability token management strategy, which employs smart contracts to register, disseminate and revoke access authorization.

#### Data access control

Nowadays, users have significant concerns around data security, whereas in traditional centralized access control mechanisms data security remains at a low level, as centralized entities may manipulate and leak user data as they wish. Some studies have introduced blockchain technology in access control to solve data security issues [105–108]. Ouaddah *et al.* [105] realized the security and anonymity of IoT data by deploying fair access in UTXOs to implement blockchain-based access control. Moreover, Le *et al.* [106] suggested an access control scheme called CapChain, which employs the anonymity of the blockchain to hide key information for data sharing and delegation to ensure data security. Also, Cha *et al.* [107] designed a novel blockchain-enabled gateway, which acts as an intermediary between users and IoT devices, thereby enhancing data security in IoT access control.

### Privacy protection

When different entities communicate with each other through wireless links, the openness of wireless transmission and mobility of wireless devices may bring many privacy issues. For example, malicious entities may intercept, relay or even tamper with the transmitted messages, which usually contain private entity identities or confidential data. Therefore, privacy protection in mobile communication networks has received increasing attention. With imbedded asymmetric encryption, blockchain is expected to provide both the privacy protection of entity identities and the privacy protection of confidential data.

#### Identity privacy

The pseudonym mechanism is often employed in blockchain to protect identity privacy by concealing the user’s identity in communication systems. In Refs. [93,109] attempts were made to use blockchain to protect privacy, where each node uses a unique public key that can be retrieved from the blockchain to communicate with other nodes. Also, Guan *et al.* [110] utilized pseudonyms in a private blockchain to hide users’ identity, where each user may create multiple pseudonyms with data related to these pseudonyms. Similarly, Lei *et al.* [111] designed dynamic key management in a vehicular communication system where users must periodically change their pseudonym set, as well as all the cryptographic materials related to this pseudonym by contacting the blockchain miners. In Ref. [112], all the activities of certificate authority are recorded in the blockchain transparently without revealing sensitive identity information of vehicles, so that public keys can be used as authenticated pseudonyms for communications. Moreover, Gai *et al.* [113] applied permissioned blockchain into smart grid networks and used a group signature technique to secure identity privacy.

#### Data privacy

Apart from identity privacy, some researches focus on the privacy protection of confidential data of users in wireless networks. The asymmetric encryption methods were used in Refs. [109,114] to encrypt user data recorded as blockchain transactions to provide privacy protection for data confidentiality. In another example, in Ref. [115], permissioned blockchain is used to retrieve the related data and manage the accessibility of data, while raw data is stored locally by each data provider in industrial IoT. Guan *et al.* [110] proposed a scheme in which the mining node is chosen according to the average consumption data and individual private data will not be disclosed for power grid communications. Different from the aforementioned mechanisms, Cha *et al.* [107] presented a blockchain-enabled IoT gateway to enhance privacy and security, by which users can manage their privacy preferences and determine whether personal data can be forwarded to an IoT device.

### Tracing, certification and supervision

With the continuously expanding scale of mobile networks and diversification of services, the demands for data traceability, device certification and information supervision become urgent, and the critical network information shall not be illegally accessed, uncontrollably manipulated and falsely spread. The existing countermeasures that use trusted third-party servers to provide data storage, device certification and tracking services suffer from privacy and security issues. Blockchain was born with features such as immutability, openness and transparency and is deemed a breakthrough solution to these concerns.

#### Tracing

Blockchain is able to provide a full range of credible records and security guarantees for tracking network entities via the mandatory operations in consensus mechanisms and smart contracts, which ensure the integrity and security of the data and transactions in blockchain. In Refs. [116,117], blockchain was proposed to enhance the traceability of IoT devices. Mitani *et al.* [118] devised an asset tracing method in which a mixed blockchain structure is adopted. Watanabe *et al.* [119] designed a new token to enhance the traceability of blockchain data. Alkhader *et al.* [120] used smart contracts to track and manage industrial transactions in manufacturing. Liu *et al.* [121] proposed an identity authentication scheme based on blockchain secret sharing and dynamic proxy and used it to track the collaborative authentication process.

#### Certification

By adopting blockchain, mobile service providers (SPs) are able to preserve and certificate devices and data transparently and reliably. Kleinaki *et al.* [122] introduced a blockchain-based certification service that uses smart contracts to seal biomedical database queries and results. Wang *et al.* [123] built a public key infrastructure (PKI) certificate system based on a permissioned blockchain and solved mutual trust problems in multicertificate-authority (multi-CA) applications. Moreover, in Refs. [124,125], blockchain was proposed to reinforce the security of device certificates in PKI systems. Cheng *et al.* [126] proposed a digital certificate system based on blockchain, implementing anti-counterfeiting and verifiable digital certificates. Xie *et al.* [127] designed a blockchain-driven certification system to achieve efficient and secure certificate queries and validations.

#### Supervision

Blockchain naturally caters to the requirements of information supervision. It was born with the capabilities of securing regulatory data and improving the efficiency of supervision and administration. Lin *et al.* [128] proposed a blockchain supervision model for e-government based on a threshold ring signature algorithm. Peng *et al.* [129] proposed a vaccine production supervision mechanism based on a two-layer blockchain. Moreover, Hassija *et al.* [130] used blockchain to create an edge computing infrastructure for workflow supervision in government bidding and significantly secured government plans and policies. Liu *et al.* [131] designed a blockchain-based autonomous transaction settlement system for IoT e-commerce, which allows all network participants to jointly supervise the settlement process.

## BLOCKCHAIN RADIO ACCESS NETWORK FOR 6G

In this section, we propose B-RAN as a unified framework of blockchain-enabled wireless communications for 6G networking. Upon the depiction of the B-RAN paradigm for 6G, we provide an in-depth discussion on the critical elements of B-RAN, including consensus mechanisms, smart contract, trustworthy access, mathematical modeling, cross-network sharing, data tracking and auditing, and intelligent networking. We also provide a prototype design of B-RAN along with the latest experimental results.

### Paradigm for 6G

Accompanying the prosperity of blockchain in the recent decade, many studies have investigated underlying blockchain technologies and their advanced applications in wireless networks, e.g. 5G and IoT, as reviewed in the previous section. However, most existing works fetch blockchain into specific scenarios separately without considering the panorama of deep and comprehensive incorporation of blockchain into wireless communications. In fact, future blockchain-empowered networking in 6G should be considered from a systematic point of view to establish an integrated system. The trust issues cannot be solved merely by introducing blockchain, but should take the complicated distrusted nature of different network layers into account to eventually form a trust foundation for 6G networks. Furthermore, most existing studies have not investigated the critical issues of blockchain in wireless environments, such as security, latency, scalability, cost, power consumption and so on. There is also a lack of mathematical models to characterize blockchain-based wireless networks as well as the corresponding experimental results. Therefore, it is imperative to address these issues and integrate advanced blockchain technologies into a unified framework for upcoming 6G.

**Figure 4.**
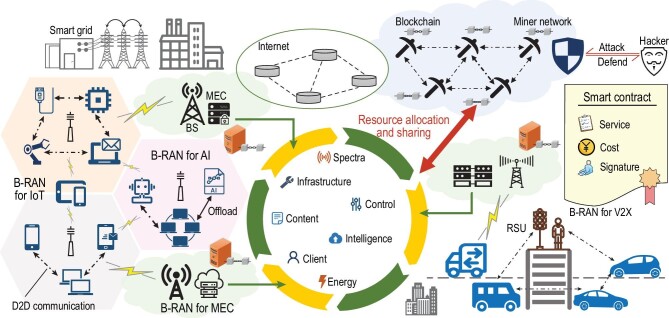
Blockchain radio access network (B-RAN): a panorama of blockchain-enabled wireless communications.

The concept of B-RAN offers a novel paradigm for large-scale, heterogeneous and trustworthy wireless networks [25]. As illustrated in Fig. 4, B-RAN acts as an open and unified framework for diverse applications to achieve resource pooling and sharing across sectors and presents an attractive solution for future 6G networks. B-RAN unites inherently untrustworthy network entities without any middleman and manages network access, authentication, authorization and accounting via trustful interactions. Via B-RAN, an MSP is established to connect different parties and facilitate resource and data sharing in a cooperative, flexible and secure way. B-RAN cannot only dynamically share computing, caching and communicating capabilities, but also deliver and spread intelligence across subnetworks. Federated-style learning can further optimize under-utilized resource allocation and network services in B-RAN. As a blockchain-as-a-service (BaaS) platform, B-RAN has distinctive security properties and is expected to provide enhanced functionalities of data exchange, privacy protection, tracking, supervision, etc.

**Figure 5.**
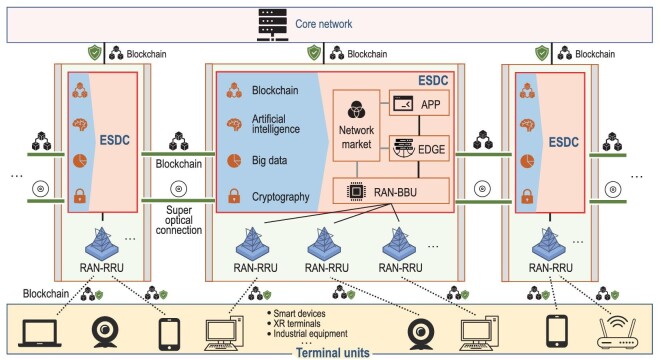
A promising architecture of 6G embedded with B-RAN.

Figure 5 demonstrates a promising architecture of 6G embedded with B-RAN. The edge super data center (ESDC) consists of powerful baseband units (BBUs) and edge servers (EDGE) and adopts a number of innovative technologies such as blockchain, AI and big data. An ESDC along with a number of remote radio units (RRUs) acts as a super base station, i.e. an enhanced counterpart of eNodeB in 5G, and supports not only wireless services but also various local applications and the network market. Within an ESDC, blockchain guarantees endogenous safety with the help of cryptography, and assisted by AI and big data, facilitates many important functionalities such as secure access control, tracking and supervision of mobile terminals. Multiple EDSCs, which may belong to different parties, are interlinked via super optical connections for high speed data exchange, and wherein, blockchain enables trusted and reliable interactions among them. In addition to ESDCs, blockchain also interconnects massive dissimilar terminal units (through RRUs), edge nodes and core networks, defining and governing their rights and obligations, and eventually achieves the network orchestration through on/off-chain smart contracts.

More specifically, in an IoT scenario, B-RAN can establish mutual trust between IoT devices and access points (APs) in a distrusted environment through the underlying blockchain, and provide a scheme for future IoT/IoE in a multioperator network [132]. This establishment of trust can avoid possibly selfish behaviors between untrustworthy devices and promote cooperation among individual IoT networks. By re-organizing multiple individual networks into a joint multi-operator network based on blockchain, B-RAN can efficiently integrate and utilize cross-network resources, such as spectra, APs, IoT devices and user data. Thus, in B-RAN, the IoT devices are not restricted to services from one subscribing SP, but can obtain resources and services across networks via effective incentive mechanisms.

Another imbedded application of B-RAN is blockchain-empowered MEC that can realize multi-party resource scheduling for an open and distributed network while providing privacy protection and data security for users. B-RAN enables direct communications between network users and MEC servers from different operators flexibly without relying on intermediary agents. The storage and computation resources among MEC participants can be fully utilized by B-RAN to reduce the vacancy and redundancy of network management and achieve the efficient configuration of resource sharing and scheduling.

### Consensus mechanisms

As the survey in the section entitled ‘Mining and consensus’ demonstrates, PoW exhibits strong robustness at the expense of great resource consumption. Since mobile devices are resource constrained, traditional consensus mechanisms (e.g. PoW and its variants) are not suitable in the mobile environment. Also, the confirmation delay is often unbearable for latency-sensitive wireless services. The drawbacks of great resource consumption and high latency become the major obstacles of traditional consensus mechanisms in a mobile environment.

Besides the potential applicability of low-cost consensus protocols, such as proof of stake (PoS) and proof of activity (PoA), an identity-based consensus mechanism named PoD [25] was developed for B-RAN. Given the fact that B-RAN is comprised of a tremendous number of devices, the PoD utilizes a unique hardware identifier (ID) that is commonly used to distinguish different devices. Based on the unique ID, every device only needs to perform the hash query once for each slot. The device that obtains a hash query less than the target threshold will be granted as the winner of the current slot. PoD significantly reduces resource consumption by restricting the number of hash operations. In this case, the uniqueness of the ID is crucial to the safety and effectiveness of PoD. To achieve this, we should introduce and use more secure features as identifiers, e.g. location, radio frequency (RF) fingerprinting, hardware security module (HSM). As an example, RF fingerprinting utilizes the imperfections of transmitter hardware to construct a unique fingerprint that identifies the device. Moreover, we can embed the HSM into the devices in B-RAN to prevent ID forgery and counterfeiting. As the unique device ID and other indispensable information is put into the device’s HSM, the users can only perform verifications without modifying the information in the HSM. Attackers can hardly alter the built-in device ID safeguarded by the HSM physically or digitally. The HSM may even erase the key information and render itself permanently inoperable if misbehaviors are detected.

In addition, a novel satellite-aided blockchain consensus protocol was devised in Ref. [133]. It makes full use of the advantages of wide coverage and ubiquitous connectivity to innovate the consensus protocol and can help construct a highly scalable space-terrestrial blockchain structure. In each round of this consensus, the satellite is responsible for periodically generating oracles and multicasting them to the terrestrial blockchain network. An oracle is a random number used to select the only winning miner in that round, who has the right to create the unique and valid new block and broadcast it to other miners via the blockchain network. This method of selecting the winner does not require massive hash queries, thus greatly reducing the energy consumption during the consensus process. The simulation results in Ref. [133] show that the proposed consensus protocol can achieve higher throughput than PoW while maintaining the same security as PoW. In addition, the delay of terrestrial P2P networks is usually long-tailed due to the large number of hops [134], while the propagation delay of satellite communication is almost fixed and more controllable [135,136]. Therefore, this consensus protocol can also be an option for B-RAN in the space-terrestrial 6G networks.

Moreover, in consensus mechanisms, one can also construct useful tasks instead of requiring participants to perform meaningless hash queries. For instance, large-scale resource allocation and scheduling in B-RAN is such a suitable mining task. Borrowing the principle of proof of learning (PoL) [31], participants in B-RAN can deploy diversiform intelligent algorithms to provide solutions to these tasks as a machine learning competition. The maintainer who offers the best solution to the scheduling scheme will be elected as the winner for the next round. Such machine learning competitions could be employed to provide solutions for manifold, complex tasks and optimize various schemes in B-RAN.

### Smart contract

The underlying blockchain and mechanisms in B-RAN guarantee system security and efficiency for resource sharing, data exchange and user access. The verifiable software codes in smart contracts ensure the consistent and automatic program execution for these services across the B-RAN network, and prevent backdoor viruses from being planted in the B-RAN system. Here, we introduce two smart-contract-based mechanisms for enhancing the security and efficiency of B-RAN.

Fast smart contract deployment (FSCD) is an advanced mechanism proposed for accelerating and securing the service in B-RAN [137]. By implementing the concept of template in smart contracts, the root contract in FSCD defines the service terms in detail, which is later automatically applied to all services. FSCD can effectively validate and trace service requests. Furthermore, it prevents forged and malicious requests from being accepted by blockchain, and thus reduces the potential risks involved in service request procedures.

Moreover, the hash time locked contract (HTLC) [138] can be utilized in B-RAN to enforce the fair resource exchange between SPs and clients. The HTLC allows users to carry out trust-free payments outside the blockchain in an ‘off-chain’ payment channel by forming a ‘restraint’ between two transactors. Because of the ‘restraint’, the breaching party is doomed to play its unprofitable role in the HTLC-based resource exchange. In this way, the HTLC credibly reduces the risks (e.g. identity spoofing risks) between SPs and clients and enhances the safety of resource trading.

Although B-RAN demonstrates its security and efficiency, a few issues remain unsettled. B-RAN is an MSP coordinating a host of networks or subnetworks, but approaches to safely isolate private information between them are still being investigated. Also, pre-computing attacks are difficult to prevent in PoD, where an attacker can use future timestamps to find a valid block in advance. Robust and efficient smart contract designs are imperative for conducting automatic penalties of violations.

### Trustworthy access

It is expected that the 6G network will contain a massive number of heterogeneous devices that belong to multiple untrustworthy parties. These devices may compete for limited resources for self-interest and simply ignore the pre-defined protocols, leading to a possible tragedy of the commons. Specifically, for grant-free access through shared links, such as IoT uplink, massive devices share a common access link without requesting permissions or dedicated resources. Because of the absence of trust, a selfish device may deliberately shorten its backoff period in random access to reduce access latency. As the number of selfish devices increases, there will be disastrous congestion in the network, which is named the Rogue's Dilemma [132].

**Figure 6.**
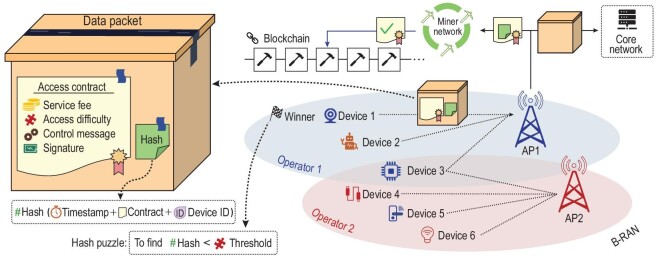
The illustration of Hash Access (adapted from Ref. [132] with permission from IEEE).

To eliminate mistrust among client devices and address the Rogue's Dilemma in grant-free scenarios, a trustworthy access scheme named Hash Access was proposed in Ref. [132], along with its mathematical analysis and evaluation [139], within the B-RAN framework. As shown in Fig. 6, each device is required to solve a hash puzzle by finding a hash value below a given threshold before transmitting packets. Otherwise, the device is denied access in the current slot. The hash puzzle is formulated by the current timestamp, its unique ID and the access contract. Owing to the pre-image resistance of the hash function, the answer to a hash puzzle can be easily verified but hardly forged. It is almost impossible for a rogue device to generate a fake hash value. Therefore, an enforced random backoff is embedded in the Hash Access scheme to reduce collisions, which can hardly be skipped by any device. In this way, the Hash Access scheme enforces devices to obey the rule of access, so as to prevent selfish behavior of rogue devices and establishes trust between client devices. In addition, the threshold in a hash puzzle determines how hard it is for each device to access, which can be adjusted accordingly to control the traffic in B-RAN. Furthermore, Hash Access ensures that the uplink resources are shared fairly, which promotes multi-party cooperation and helps to integrate the cross-network resources and efficiently offload traffic.

In addition to the misbehavior of malicious IoT devices discussed above, there are many other device access security risks in different layers of a wireless network as investigated in the section entitled ‘Secure access control’. Secure and robust device access control approaches for 6G networks are to be developed. B-RAN provides an ideal platform to integrate various access methods and protocols into the 6G architecture from a systematic point of view via establishing cooperative trust and security among heterogeneous entities that may have potential security risks.

### Mathematical modeling

Despite the fact that blockchain-based networking has come into focus, works regarding mathematical modeling and fundamental analysis are rather limited. A considerable amount of intractable issues remain unsolved. The existing works have not assessed the impact of decentralization on RANs after introducing blockchain, which, however, should be analytically characterized and accurately quantified. Very few works have noted that service latency will be a crucial problem for B-RAN as a price of decentralization, the length and controllability of which, unfortunately, is still unknown. As another critical aspect of B-RAN, security has not been thoroughly looked into yet. Therefore, an analytical model is urgent to explore the characteristics of B-RAN (such as latency and security) and to provide insightful guidelines for real-world implementations further.

In Ref. [140], we made an original attempt to mathematically model B-RAN and analytically characterize its properties and performances. Specifically, we modeled the block generation via a Poisson process and verified it by real data. We then established a queueing model embedded by a continuous time-homogeneous Markov process for B-RAN. Based on the queueing model, we presented a general state transition graph, evaluated the service-level latency in the average sense and revealed the impact of critical parameters on the B-RAN latency by further deriving tight upper and lower bounds. Meanwhile, we assessed the security level of B-RAN by considering the attacker’s strategy.

**Figure 7.**
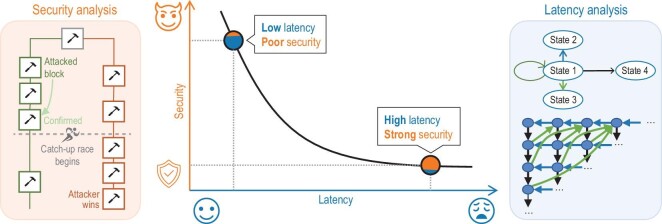
Mathematical model of B-RAN and the latency-security trade-off.

From the above analysis on latency and security, we discovered an inherent relationship between them, which can be described by the latency-security trade-off curve shown in Fig. 7. On the one hand, the request latency of B-RAN is quasi-linear to the block generation time, and it grows as the number of confirmations for verification or the block generation time increases. On the other hand, more confirmations are required to reduce the probability of a successful attack. The confirmation number becomes the key factor in balancing the service latency and system security in B-RAN and shall be carefully selected. It is worth pointing out that such a trade-off characterizes the achievable performance of B-RAN comprehensively. Our analytical model in Ref. [140] provides meaningful inspirations for designing blockchain-based wireless networks with both enough security against malicious miners and affordable access latency.

### Cross-network sharing

Rather than an oligopoly, B-RAN recruits a large number of SPs and clients, enabling significant resource sharing across subnetworks. As the number of clients increases, more SPs will join out of economic incentives. With more SPs, the improved quality of service (QoS), in turn, attracts more clients, creating a positive feedback loop based on network effects. As well as recruiting more SPs and clients, B-RAN functions as an MSP, a platform letting multilateral groups (SPs, clients, and others in B-RAN) on board and enabling direct interactions between them. Such multi-sidedness leads to various sources of revenue in B-RAN. These assets or services from different participants are commodified and put into a vast resource pool in B-RAN and then virtualized via smart contract, promoting further resource sharing and pooling. Here we summarize several important kinds of resources in B-RAN.

1. *Spectra.* The spectra in B-RAN are virtualized as digitized spectrum assets. A spectrum asset can be defined as an exclusive usage right within the assigned time duration to transmit on a frequency range in a given coverage area. The utilization of spectrum assets from multiple spectrum holders will be more efficient and flexible than that of a single-operator network.
2. *Infrastructures.* Infrastructures in B-RAN include all types of APs and base stations (BSs), MEC and cloud servers, backhauls, etc. Infrastructures belonging to different hosts can be shared across SPs and individuals via computation offloading, data storage, or network access services. Such coordination can significantly reduce utilization redundancy and improve network efficiency.
3. *Devices.* Mobile client devices can be exploited to collectively gather data and extract information for large-scale services of SPs or other applications, while various IoT/IoE devices can be involved in crowdsensing. Also, the growth of client numbers will attract more SPs to B-RAN and further achieve economies of scale. Client resources can benefit multilateral groups and motivate B-RAN to continue growing.
4. *Content.* Content in B-RAN can be media files, software, documents, applications, live streaming, etc. Not only will SPs provide content delivery services, but clients will also be encouraged to participate in providing content. Under the security and privacy protection of B-RAN, the content will be tamper-proof and accessed only by authorized users in delivery.
5. *Control.* Control rights over multiple devices can be viewed as resources in B-RAN. Network devices with functionality for packet forwarding or smart devices used as home appliances will function at the control command of all permitted users with a sharing key. With the underlying blockchain, a more trustworthy and reliable control can be implemented in B-RAN.
6. *Energy.* Energy in B-RAN is usually electricity energy coming from fossil or renewable energy resources. Devices with sufficient idle energy can conduct a discharging operation for energy supply demands of other devices and obtain payment. Energy prosumers and consumers can interact with each other securely and fairly in B-RAN.
7. *Intelligence.* Intelligence in B-RAN represents trusted learning models and capabilities, such as computing, caching and communicating, to perform learning algorithms. In B-RAN, learning models can be performed in a federated manner on heterogeneous devices and the data confidentiality of such collaborative learning process will be ensured. Reliable intelligence will be distributed efficiently in B-RAN.

**Figure 8.**
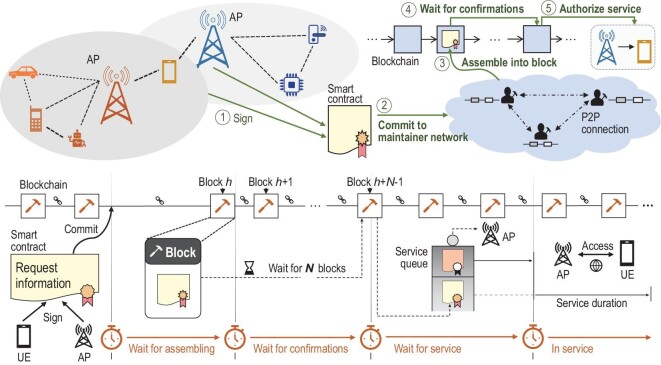
The basic workflow of B-RAN.

An example B-RAN service process that has five steps is presented in Fig. 8. The following is the basic procedure of how B-RAN works to help a user equipment (UE) request services from an SP.

1. In preparation for access, the UE and SP should first enter a service-level agreement (SLA) containing details including service types and compensation rates. The service terms and fees will be explicitly recorded in a smart contract authorized by the digital signatures of both sides.
2. The smart contract is committed to the blockchain network, waiting for the blockchain maintainers to verify its validity.
3. The blockchain network maintainers finish the verification of the smart contract and will record it in a new block after the current round of consensus is reached.
4. The block is accepted into the main chain after a certain amount of blocks as confirmations built on top of it. The smart contract is then confirmed secure and qualified to enter the service queue.
5. When finishing the services of preceding requests, the SP will deliver the access service to the UE according to the request information in the smart contract.

It is worth noting that the superiority of B-RAN regarding efficiency lies in the network pooling principle that requires flexible offloading and sharing between subnetworks. In the previous example, the UE has established trust with SPs via the procedure in Fig. 8, and the UE can thus access and use resources pooled by other SPs in B-RAN. The trading and roaming charges would be calculated and settled periodically by smart contracts. In this case, the maintainers in B-RAN can use some intelligent algorithms to allocate and distribute the pooled resources for higher network efficiency. As a result, mobile devices may access suitable SPs belonging to the subnetworks that likely provide higher-quality coverage for the UEs in their current locations.

### Data tracking and auditing

In the era of big data, the expeditious growth of data has brought many challenges for enterprises, societies and governments. Data breaches occur more frequently than ever and are now causing serious security risks [141]. Wireless networks, due to openness and mobility, are more vulnerable to data leakage and malicious intrusion. The increasing demand of data security and user privacy calls for necessary data tracking and auditing approaches to detect data leakage and prevent unauthorized access and usage of sensitive data. Some countries and organizations have issued relevant regulations on data usage to curb data leakage and ensure data tracking and reliable auditing. For instance, the European Union released the General Data Protection Regulation (GDPR) in 2018 [142], and the United States issued the National Security and Personal Data Protection Act (NSPDPA) of 2019 [143] on data protection and transmission.

The current network data tracking and auditing methods are mostly based on deep packet inspection (DPI) [144] and traffic analysis [145]. DPI-based schemes often require manual discoveries of traffic characteristics and a large amount of data processing, and traffic analysis may also suffer from real-time performance and deployment efficiency, which make them difficult to scale with the rapid growth of big data. Note that data marking techniques can also be used to trace network flows, e.g. in Refs. [146,147] two watermarking schemes were designed for data traceback and analysis in mobile networks. Recently, several studies [148,149] have shown the feasibility of using blockchain to fulfill or facilitate data tracking and auditing for cryptocurrency, health care and food supply chains. Yet, blockchain-enabled data tracking and auditing approaches for wireless networks are still open.

**Figure 9.**
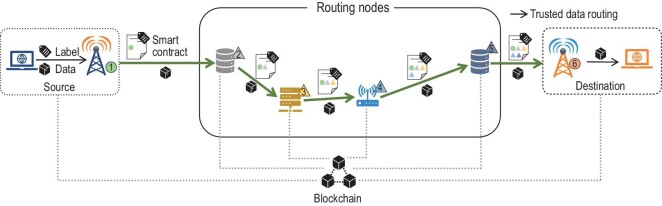
Data tracking and the auditing scheme in B-RAN.

In B-RAN, data are delivered through several relay paths via a number of devices and infrastructures. A blockchain consisting of entities from multiple parties can record the routing path of data in a trustworthy and transparent way and thus is suitable for tracking and auditing data. By incorporating the data marking technique, a data tracking and auditing scheme in B-RAN is designed and shown in Fig. 9. The scheme lets routing nodes, which may be from various manufactures and operators, participate in B-RAN and report their sight of routing data to the blockchain via smart contracts. To protect the authenticity of data and its origin, the data source is required to generate an immutable digital label for its transmitting data using the trusted platform module (TPM) inside its device. The smart contract records the data label and information of the source. In addition, each relay node has to add its digital signature and commit the latest contract to the blockchain, which forms a trusted routing path consisting of smart contracts. Thus, the relaying path of data is jointly audited by the multiple parties in B-RAN, and can hardly be forged or modified since the data paths are secured by the blockchain. Also, this scheme can facilitate the data review and violation monitoring conducted by regulatory authorities.

### Intelligent networking

B-RAN can provide an intelligent resource provisioning mechanism to manage network resources in a distributed learning approach. The maintainers in B-RAN can monitor the resource conditions and optimize under-utilized resource assignment through machine learning technologies. As discussed in the section entitled ‘Consensus mechanisms’, the intelligent consensus of B-RAN provides credible prospects for spectra and infrastructure sharing, which helps network operators better serve customers. Additionally, energy trading, computation offloading and storage sharing can also be achieved in B-RAN.

Because of its distributed nature, B-RAN inherently supports federated-style learning. B-RAN can exchange trusted models and share the capabilities that are necessary for machine learning, resulting in high efficiency of federated-style learning. As the underlying blockchain establishes multifold trust relationships for B-RAN, network entities can share intelligence in an open, compatible manner. Also, B-RAN can track the entire process of the data procedure for trust considerations, enhancing the interpretability and credibility of machine learning. Therefore, B-RAN will promote the development of federated-style learning and eventually achieve strong trusted intelligence.

In turn, B-RAN also improves network quality and provides intelligent services. In response to clients’ requests, B-RAN maintainers can schedule and assign services via a distributed learning approach, leading to an adaptive smart network. B-RAN can collect users’ data to adjust real-time service quality, while service scheduling is performed as a federated learning task among different network entities. Clients can also select and use the appropriate signal transmission medium such as millimeter wave, visible light, infrared and terahertz wave, depending on the specific scenarios.

Also, B-RAN can monitor the network status and utilize distributed learning to avoid traffic congestion and achieve fast fault locations. Via deep learning from historical data, B-RAN can predict the trend of traffic and prevent traffic congestion. To achieve a balance between limited network resources and service quality, data packets need to be prioritized to ensure optimum experience for network operators and users. This can be achieved by utilizing AI technology. With the proliferation of devices and users, fault location has ushered in new challenges due to the spatial correlation of alert messages and the interaction between failures via machine learning algorithms, such as the learn vector quantization neural network [150] and the deep neural evolution network [151].

### Prototype design

**Figure 10.**
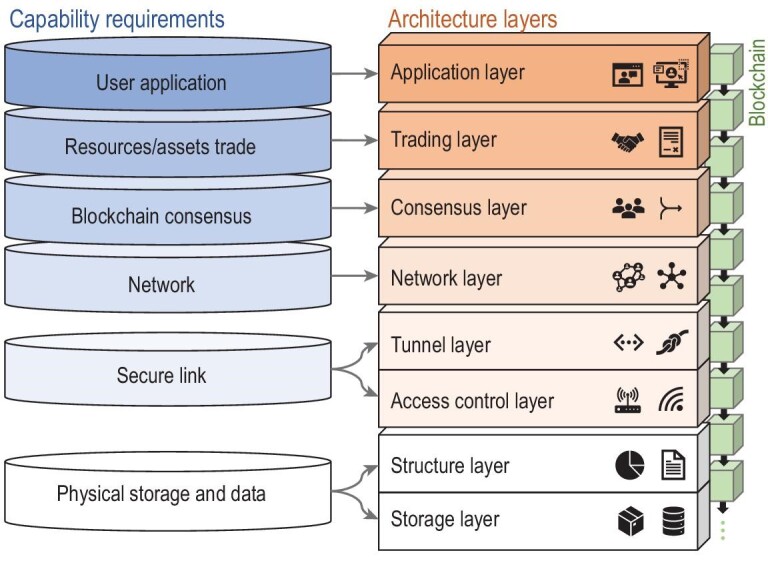
Basic capability needs and architecture layer design of B-RAN.

In order to implement the proposed design and better test its performance, we assess the basic capability needs in B-RAN and construct a corresponding prototype. The capability needs cover six aspects, including physical storage and data structure, secure link, network, blockchain consensus, resources and assets trading, and user applications. Based on the evaluated needs, we further design the corresponding architecture layers for the prototype. We introduce the access control layer, tunnel layer, consensus layer and trading layer into our architecture. Apart from these, we also use several traditional network system layers (i.e. storage layer, structure layer, network layer and application layer) to support basic operations of our prototype. Considering the unique application scenarios of B-RAN, we further incorporate the mechanisms FSCD, HTLC and Hash Access into the architecture to improve the overall performance of the prototype system. We now provide more details about the basic capability needs and the design of the architecture layers shown in Fig. 10.

1. *Physical storage and data.* All the blockchain data or information associated with B-RAN such as the ordered transactions, digital actions and cryptographic keys form the structure layer. Such data are stored by servers or any active electronic devices in the storage layer. Not all devices will store a full copy of a blockchain, as some mobile devices can store partial information as lightweight nodes.
2. *Secure link.* A secure link between different sides is established in B-RAN. By leveraging the principle of HTLC in the tunnel layer, the payment channel between terminals and APs is trust free. Transmission reliability is also ensured by flow control and error detection in the access control layer. Link duration, fees and even physical transfer media are regulated by smart contracts.
3. *Network.* Subnetworks with various structures in B-RAN form the network. The nodes and the links between them in the network layer are responsible for nodes discovery and communication, and synchronize with each other to maintain the distributed network.
4. *Blockchain consensus.* The consensus in B-RAN is responsible for generating and validating the blocks and ensuring participants reach a consensus about the broadcast transactions. All the maintainers in B-RAN follow the rules in the consensus layer to determine which block will be added to the ledger.
5. *Resources/assets trading.* The execution of resources/assets trading is performed with the underlying rules in B-RAN to keep the fairness between SPs and clients. Such fairness is ensured by smart-contract-enabled service-level agreements in the trading layer.
6. *User application.* User applications on top of the blockchain are designed for clients to interact with the blockchain and smart contract. Application programming interfaces in the application layer will also be provided for developers to implement some other desired functionality apart from access services.

### Experimental results

In this section, several experiments are presented to evaluate the performance of B-RAN under varied circumstances. We implement the prototype using Python and test its packed binary executables on a cluster of single-board computers running under the same local area network. The data are collected automatically through a pre-designed script. Furthermore, to verify the B-RAN performance, we take normal RANs and PoW-based blockchains as the benchmark, and compare the collected data with those of existing schemes. In the following, we mainly introduce and analyse the results of three experiments regarding service latency, resource utilization and request processing.

**Figure 11.**
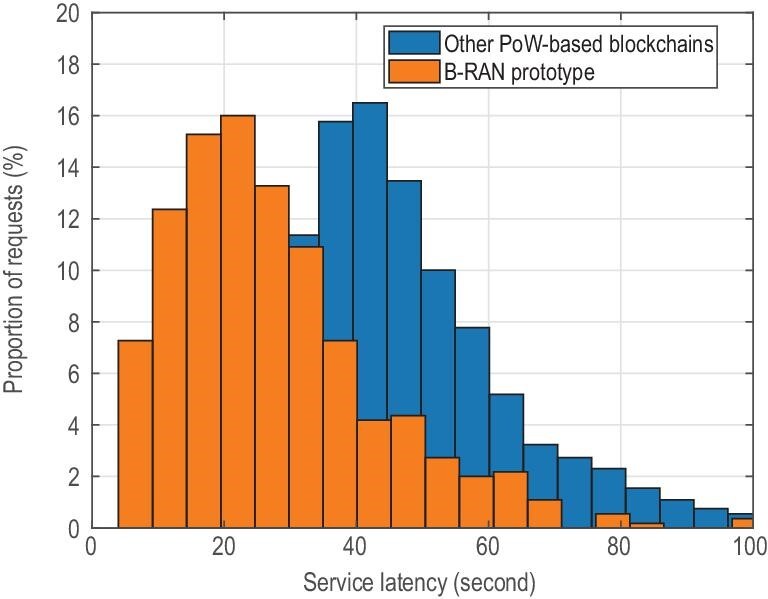
Comparison of the service latency distribution of the B-RAN prototype and other PoW-based blockchains (adapted from Ref. [137] with permission from IEEE).

Figure 11 compares the service latency distribution of the PoW-based B-RAN prototype with that of other PoW-based blockchains (e.g. Bitcoin and Ethereum). In the experiment, the block time is set to 10 s, and two blocks are needed as blockchain confirmations. As shown in Fig. 11, B-RAN has significantly lower service latency than ordinary PoW-based blockchains. In addition, under a variety of circumstances, service latency in ordinary blockchains usually lasts for several minutes. By equipping the FSCD mechanism, B-RAN nearly halves the required service period, and shortens the service latency down to seconds. Moreover, the shortened service latency may have a positive impact on the user satisfaction of B-RAN, and further improve the system security (see the section entitled ‘Cross-network sharing’).

**Figure 12.**
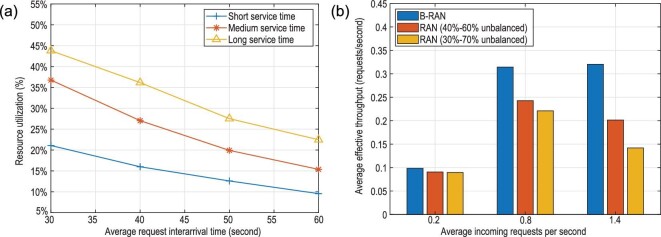
Resource utilization and throughput evaluation based on the B-RAN prototype. (a) The influence of the average request interarrival time on resource utilization under different service times (adapted from Ref. [137] with permission from IEEE). (b) Effective throughput of B-RAN and two other unbalanced RANs at different traffic intensities.

Figure 12a shows the influence of the average request interarrival time on the resource utilization of B-RAN, where the resource utilization is measured by the ratio of the busy time to the available time of the links, and indicates how effectively the links are utilized against their availability. It can be clearly observed that, whatever the service time, the decrease in the average request interarrival time always leads to an obvious increase in the resource utilization. Furthermore, by comparing the trends of three different service time settings, one may conclude that, when the average service time over a last time period is high, or is presenting an upward trend, the higher request intensity (or more engaged users) will bring a greater growth to the resource utilization than shorter service time scenarios. Thus, the further growth of resource utilization is also closely related to the expansion of network scale and the increase in user engagement of B-RAN.

Figure 12b compares the average effective throughput per subnetwork of B-RAN and the other two ‘unbalanced’ RANs at different incoming request frequencies. In this experiment, both B-RAN and RAN are composed of two subnetworks; however, unlike the former, RANs are usually composed of two independent subnetworks that have different traffic loads. As can be seen in Fig. 12b, the RANs tested in our experiment share the same traffic load as B-RAN, but the requests are nonequally allocated to their subnetworks with ratios of 40% to 60% and 30% to 70%. The experimental results show that the average effective throughput of B-RAN is the best in the three simulated scenarios. Because of the special capabilities of integrating subnetworks and balancing traffic loads, B-RAN has a much higher effective throughput than the other two ‘unbalanced’ RANs when the incoming request rate is higher.

The simulation demonstrates the advantages of B-RAN in terms of low service latency, high resource utilization and prominent load balance. The remarkable decrease in service latency gives credit to the FSCD mechanism that effectively increases the contract deployment rate and guarantees that contracts and transactions are recorded in blockchain in one step. In addition, the reduction in the contract deployment delay extends the service time within a fixed period, consequently improving resource utilization. Moreover, since B-RAN consists of multiple subnetworks, its request processing rate far exceeds that of a single network, and the increasing number of subnetworks accommodated by B-RAN could also bring a more significant improvement in throughput performance.

## CONCLUSION

In this paper we have identified the critical trust-related issues in wireless networks that impede the evolution of current 5G networks towards more efficient and secure 6G networks. Upon a brief introduction of the fundamentals of blockchain, we comprehensively investigated the recent research works on applying blockchain to wireless networks from several aspects, including resource sharing, trusted data interaction, secure access control, privacy protection, tracing, certification and supervision. Then, a unified B-RAN framework was proposed as a trustworthy and secure paradigm for 6G networking by utilizing blockchain technologies with enhanced efficiency and security. We elaborated on the critical elements of B-RAN, such as consensus mechanisms, smart contract, trustworthy access, mathematical modeling, cross-network sharing, data tracking and auditing, and intelligent networking, and provided the prototype design of B-RAN along with the latest experimental results.
